# Spring Diseases

**Published:** 1883-02

**Authors:** 


### SPRING DISEASES.

THE complaint of "lassitude" is almost universal as spring advances, and those who have reached fifty years, can well remember the old time custom of taking something to "purify the blood," to "thin the blood," as regularly as the season of spring returned; and even now, the failing in appetite and "falling off" in flesh corroborate the idea in the unthinking, that they must take something, and forthwith "bitters" are prepared, and these bitters, being nothing less than some herb or root put into a bottle of whisky, are the means of initiating multitudes into habits of drunkenness. The more elevated and refined of cities, use various kinds of wines, and too often recommend their children to do the same, to end in drinking vulgar gin, or in secretly chewing opium.

But there is a better way and a safer. The decline of appetite in spring is not the symptom, or the effect of disease, it is as it were the wise forethought of a sleepless instinct, which puts out its blind feelers ahead to clear away danger. Instinct, that wonderful, impalpable thing, the agent of Almighty power, the instrument of love d[illegible], its lesson is, that the body does not require so much food, hence the desire for it is taken away; and if men could only be induced to read that lesson aright, to practice it by simply eating according to the appetite, by not going to the table if they did not "feel like taking anything," and then resolutely wait until the next meal, and at no time eating an atom, unless there was a decided desire for it,—if such a course were judiciously pursued, the spring time would be to us a waking up to newness of life, as it is to the vegetable world. But instead of thus co-operating with our instincts, we "take something," bitters, pills, any thing that any body advises as good for "whetting up the appetite." It acts like a charm, we speak loudly in its praise, and a dozen more are induced to follow the example. But soon the bubble bursts. Nature was only drugged, her voice was hushed, only to wake up a little later to find her ward prostrated by serious, and as to old persons, often fatal sickness. To avoid spring diseases then, abate the amount of food eaten at least one third, and work or exercise with a proportionate deliberation.

### A PATENT AND PATTERN MOTHER.

A physician of superior abilities in a neighboring state had a daughter of sweet seventeen, whose health was not as good as was desired, and not wishing to give her medicine, he made arrangements for having her travel leisurely through New England for several months, as the most likely means of her restoration. At the same time her mother obtained a supply of various patent medicines, and placed them in the travelling trunk, *with private instructions* to take them while travelling. The daughter to accommodate herself and her father, took the journey, and in obedience to the mother, took the physic, and as a matter of course, is—still an invalid. The father remains in ignorance of the cause of the failure of beneficial results from his prescription.

Lesson.—*The child who privately does a thing for one parent, known to be contrary to the wishes of the other, and the parent who can counsel it, are equally guilty of a violence against domestic rule, which will not cease to bear the pernicious fruits of deception and discord, to the latest hour of their lives.*

